# Identification of polyphenols as novel neuropilin-1 cendR pocket inhibitors to block SARS-CoV-2 entry and enhance variant resistance

**DOI:** 10.1371/journal.pone.0345051

**Published:** 2026-03-23

**Authors:** Farid Ataya, Abir Alamro, Amani Alghamdi, Dalia Fouad

**Affiliations:** 1 Department of Biochemistry, College of Science, King Saud University, Riyadh, Saudi Arabia,; 2 Department of Zoology, College of Science, King Saud University, Riyadh, Saudi Arabia; Kwara State University, NIGERIA

## Abstract

Neuropilin-1 (NRP-1) functions as an essential co-receptor for SARS-CoV-2, facilitating viral entry by binding the spike protein’s C-end rule (CendR) motif in its b1 domain, yet it has received less attention than ACE2 in therapeutic development. This *in silico* study evaluates plant-derived polyphenols as potential selective inhibitors of the NRP-1 CendR pocket to disrupt SARS-CoV-2 engagement, addressing limitations of synthetic inhibitors like EG01377, which exhibit modest affinity (−5.83 kcal/mol) and potential off-target risks. High-throughput molecular docking of 10,000 phytochemicals using AutoDock Vina identified 10 polyphenols with binding affinities ranging from −9.87 to −6.63 kcal/mol, led by 6“-O-acetyldaidzin (-9.87 kcal/mol) and phloretin (-8.64 kcal/mol), forming stable hydrogen bonds and π-cation interactions with critical residues (e.g., THR-401, GLU-367 for 6”-O-acetyldaidzin; PRO-311, ILE-400 for phloretin), as visualized in Discovery Studio. Notably, four of the top inhibitors are isoflavonoid derivatives, highlighting a chemical class enrichment. Molecular dynamics simulations over 100 ns using Desmond indicated moderate complex stability (RMSD: 0.6–3.8 Å; RMSF <0.5 Å at binding site**).** ADMET-Tox profiling via SwissADME and ProTox-II revealed drug-like properties, including high gastrointestinal absorption (>70% for leads) and low toxicity (classes 4–5), though 6”-O-acetyldaidzin shows limited bioavailability due to its high H-bond acceptor count (10) and large size, suggesting need for formulation optimization. The NRP-1 b1 homology model, built with SWISS-MODEL, exhibited high fidelity (GMQE: 0.79; Ramachandran favored regions: 90.3%**).** This focused computational screening of polyphenols against NRP-1 complements prior studies and identifies candidates for experimental validation as potential SARS-CoV-2 inhibitors. Limitations include the *in silico* nature, and lack of membrane/sialic acid models, necessitating *in vitro* and *in vivo* testing against SARS-CoV-2 variants.

## Introduction

The ongoing threat of SARS-CoV-2, even in 2025 with emerging variants, highlights the need for innovative antiviral strategies, like those targeting viral entry [1]. While angiotensin-converting enzyme 2 (ACE2) remains the primary receptor, its inhibition often yields off-target effects due to ACE2’s roles in cardiovascular and renal homeostasis, and efficacy wanes against variants like Omicron sublineages [1–3]. Neuropilin-1 (NRP-1), a transmembrane co-receptor, facilitates viral entry by binding the spike protein’s C-end rule (CendR) motif in its b1 domain, (residues ~273–427 in the full protein, aligning with resolved structures in PDB 2QQK), promoting endocytosis in ACE2-expressing cells, particularly in respiratory and neural tissues [4,5]. Recent studies confirm NRP-1’s role in variant persistence, yet therapeutic targeting lags behind ACE2, with limited focus on NRP-1 in entry inhibitor research [6,7]. Synthetic inhibitors like EG01377 bind with low affinity (−5.83 kcal/mol) and exhibit vascular off-target effects, necessitating selective alternatives [8,9].

Polyphenols, abundant in plants, emerge as promising candidates for NRP-1 modulation due to their low toxicity, structural diversity for pocket binding, and reported antiviral-relevant mechanisms, including interference with viral entry and host receptor interactions [10–12]. Their aromatic rings and hydroxyl groups facilitate interactions with NRP-1’s electrostatic CendR pocket, defined by key residues such as Tyr297, Asp320, Ser346, potentially blocking spike engagement—a mechanism explored in recent reports [7,13]. Unlike ACE2 inhibitors risking hypertension, NRP-1-targeted polyphenols may preserve homeostasis while leveraging anti-inflammatory properties to counter COVID-19 hyperinflammation [14,15]. However, systematic polyphenol-focused analyses against NRP-1 remain limited, representing a gap in natural product-based antiviral research [7,16].

*In silico* methods, including docking, MD simulations, and ADMET profiling, enable efficient screening of vast libraries, predicting affinities and stabilities with >80% accuracy in validation studies [17,18]. This study employs an *in-silico* pipeline to screen 10,000 phytochemicals against a validated NRP-1 b1 model (GMQE 0.79**),** identifying high-affinity polyphenols for CendR pocket inhibition. By filling the NRP-1 research gap and connecting polyphenols’ therapeutic promise, this work advances hypothesis-generating insights for novel COVID-19 interventions, with implications for variant-resistant strategies. The focus on polyphenols is justified by their compatibility with the CendR pocket’s electrostatic features and established safety, excluding other natural product classes to maintain scope.

## Methodology

### NRP-1 b1 domain modeling

The protein sequence of human neuropilin-1 (NRP-1) was retrieved from the National Center for Biotechnology Information (NCBI) database (Accession: NP_003864.5, 923 amino acids) [19]. The b1 domain (residues ~273–427, corresponding to PDB 2QQK boundaries), critical for binding the SARS-CoV-2 spike protein’s C-end rule (CendR) motif, was modeled using SWISS-MODEL (version 2021) [20]. While experimental structures like PDB 2QQK exist, a homology model was used for consistency with prior screening workflows and uniform residue coverage. Template selection prioritized PDB 2QQK with >65% sequence coverage and >30% sequence identity to ensure high structural fidelity. Structural quality was assessed using QMEAN scoring (achieved score: −1.4) and Ramachandran plot analysis (90.3% in favored regions, 9.5% allowed, 0.2% generously allowed, 0% disallowed) [20,21]. Physicochemical properties of the NRP-1 protein, including molecular weight, isoelectric point, charge distribution, extinction coefficient, instability index, and grand average of hydropathicity (GRAVY), were calculated using the ProtParam tool on the ExPASy server (version 2021), [22] to inform subsequent ligand interaction analyses. The final model was saved in PDB format for docking preparation. The scripts for modeling are available in study and supplementary file for reproducibility.

### Molecular docking analysis

#### Ligand preparation.

A library of 10,000 phytochemicals, enriched in polyphenols (e.g., chalcones, flavones, isoflavones), was curated from PubChem (version 2021), [23], PhytoHub [24], and ZINC15 databases [25], selected based on their reported antiviral and anti-inflammatory properties**.** Three-dimensional (3D) structures were retrieved in SDF format and subjected to energy minimization using the MMFF94 force field in Discovery Studio 2021 [26]. Ligands were protonated at physiological pH (7.4) using dominant states, acknowledging that tautomers were not exhaustively sampled. Canonical SMILES notations were generated using Open Babel (version 3.1.1), [27] and validated for chemical accuracy. The synthetic NRP-1 inhibitor EG01377 (PubChem CID: 135398512) was included as a control for benchmarking.

### Receptor preparation

The NRP-1 b1 domain model was prepared for docking by removing water molecules, ions, and pre-existing ligands using Discovery Studio 2021 [26] and PyRx (version 0.8) [28]. The receptor was energy-minimized using the GROMOS 96 force field in SWISS-MODEL to optimize side-chain geometries and charge states at pH 7.4. The CendR pocket was designated as the docking target based on structural data from PDB 2QQK and prior studies identifying its role in spike protein binding [4]. Partial charges were assigned using the Gasteiger method in AutoDockTools (version 1.5.7) [29] to ensure accurate electrostatic interactions.

### PyRx docking

Molecular docking was conducted using PyRx (version 0.8) with the AutoDock Vina algorithm (version 1.1.2) [30] to identify optimal ligand conformations by minimizing binding free energy. The docking grid was centered on the CendR pocket (coordinates from PDB 2QQK), with dimensions of 25 × 25 × 25 Å, encompassing key residues. An exhaustiveness parameter of 8 was applied to balance computational feasibility and sampling, acknowledging this as a limitation for ultra-large libraries without decoy sets for false-positive control. Binding affinities were evaluated using Vina scores (with standard deviations across poses reported**),** where lower values indicate relative favorable interactions. Docking accuracy was validated by redocking a known NRP-1 ligand (VEGF-A mimetic) to confirm grid reliability (achieved RMSD <1.5 Å). Protein–ligand interactions, including hydrogen bonds (noting those >3.5 Å as weak**),** π-cation, and hydrophobic contacts, were visualized in 2D and 3D using PyRx and Discovery Studio Visualizer [26].

### ADMET-Tox profiling

Pharmacokinetic and toxicity profiles of selected phytochemicals were evaluated using ADMET analysis. Canonical SMILES were submitted to SwissADME (version 2021) [18] to assess parameters including gastrointestinal absorption, blood–brain barrier permeability, cytochrome P450 inhibition (e.g., CYP3A4, CYP2D6), and compliance with Lipinski’s Rule of Five (molecular weight <500 Da, hydrogen bond donors <5, acceptors <10, LogP < 5). Acute oral toxicity was predicted using ProTox-II (version 2021) [31], classifying compounds into six toxicity classes (1: highest toxicity, LD50 < 5 mg/kg; 6: non-toxic, LD50 > 5000 mg/kg) based on median lethal dose (LD50). Additional toxicity endpoints, including hepatotoxicity, carcinogenicity, and mutagenicity, were evaluated to ensure safety. Results were compiled for prioritization of compounds for further analysis. PAINS alerts were checked, noting potential false positives for polyphenols.

### Molecular dynamics simulation

The stability of selected phytochemical–NRP-1 b1 domain complexes was assessed using 100-nanosecond molecular dynamics (MD) simulations performed with Desmond (version 2021−4) [32]. The OPLS3e force field was applied within an explicit TIP3P water model, using a 10 Å orthorhombic box neutralized with Na ⁺ /Cl⁻ ions to achieve a physiological ionic strength of 0.15 M [33]. Ligand–receptor complexes were prepared using Maestro’s Protein Preparation Wizard (version 2021−4), correcting bond orders, adding hydrogens, and optimizing hydrogen bonds at pH 7.4. The system was configured using the System Builder tool, maintaining 310 K and 1.01325 bar pressure via the NPT ensemble with a Nosé-Hoover thermostat (relaxation time: 0.1 ps) and Martyna-Tobias-Klein barostat (relaxation time: 2.0 ps). Simulations were conducted with a 2 fs time step, recording trajectories at 100-picosecond intervals. Stability was evaluated through Root Mean Square Deviation (RMSD) of protein Cα atoms and ligands, Root Mean Square Fluctuation (RMSF), Radius of Gyration (rGyr), Solvent Accessible Surface Area (SASA), and hydrogen bonding interactions, with convergence assessed over the final 100 ns using Desmond’s Simulation Interaction Diagram tool [34]. No membrane or sialic acid models were included, a limitation for biological realism.

## Results

### Prediction of the 3D structure

**Fig 1:**
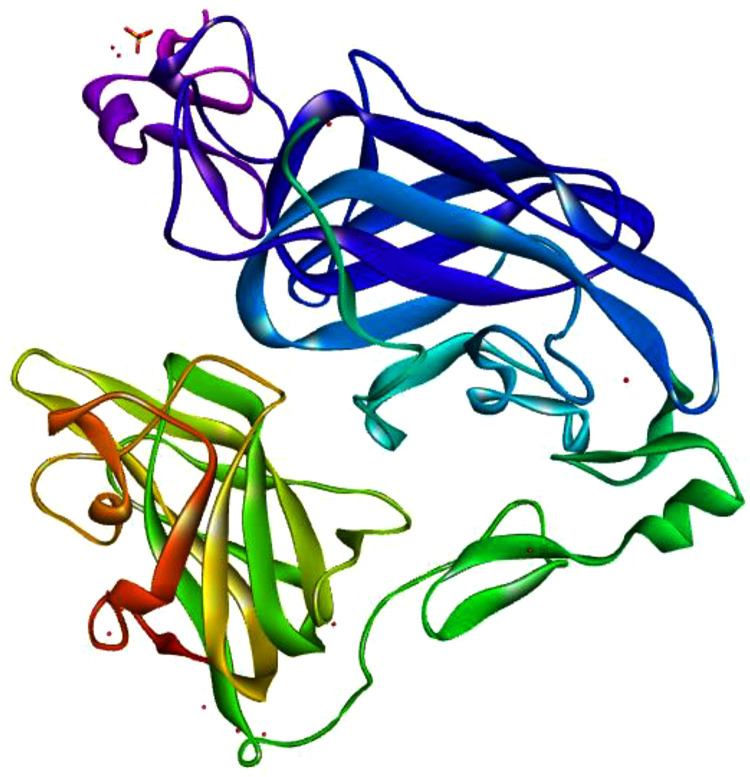
3D Structure of human neuropilin-1 b1 domain.

The protein sequence of human neuropilin-1 (NRP-1) was retrieved from the National Center for Biotechnology Information (NCBI) database (Accession: NP_003864.5, 923 amino acids). BLASTp analysis confirmed a query coverage of 100% and sequence identity of 99.9% with known NRP-1 sequences, ensuring high sequence reliability for modeling**. The b1 domain (**residues ~273–427**),** critical for SARS-CoV-2 spike protein C-end rule (CendR) motif binding, was modeled using SWISS-MODEL [20]. The resulting model was saved in PDB format and visualized using PyRx (Table 1, Fig 1).

**Table 1:** Protein sequences of human neuropilin-1 b1 domain were obtained from the NCBI.

Sr.	Protein	Length	Sequence of human neuropilin-1 b1 domain
1	Human neuropilin-1 b1 domain [NP_003864]	923	MERGLPLLCAVLALVLAPAGAFRNDKCGDTIKIESPGYLTSPGYPHSYHPSEKCEWLIQAPDPYQRIMINFNPHFDLEDRDCKYDYVEVFDGENENGHFRGKFCGKIAPPPVVSSGPFLFIKFVSDYETHGAGFSIRYEIFKRGPECSQNYTTPSGVIKSPGFPEKYPNSLECTYIVFVPKMSEIILEFESFDLEPDSNPPGGMFCRYDRLEIWDGFPDVGPHIGRYCGQKTPGRIRSSSGILSMVFYTDSAIAKEGFSANYSVLQSSVSEDFKCMEALGMESGEIHSDQITASSQYSTNWSAERSRLNYPENGWTPGEDSYREWIQVDLGLLRFVTAVGTQGAISKETKKKYYVKTYKIDVSSNGEDWITIKEGNKPVLFQGNTNPTDVVVAVFPKPLITRFVRIKPATWETGISMRFEVYGCKITDYPCSGMLGMVSGLISDSQITSSNQGDRNWMPENIRLVTSRSGWALPPAPHSYINEWLQIDLGEEKIVRGIIIQGGKHRENKVFMRKFKIGYSNNGSDWKMIMDDSKRKAKSFEGNNNYDTPELRTFPALSTRFIRIYPERATHGGLGLRMELLGCEVEAPTAGPTTPNGNLVDECDDDQANCHSGTGDDFQLTGGTTVLATEKPTVIDSTIQSEFPTYGFNCEFGWGSHKTFCHWEHDNHVQLKWSVLTSKTGPIQDHTGDGNFIYSQADENQKGKVARLVSPVVYSQNSAHCMTFWYHMSGSHVGTLRVKLRYQKPEEYDQLVWMAIGHQGDHWKEGRVLLHKSLKLYQVIFEGEIGKGNLGGIAVDDISINNHISQEDCAKPADLDKKNPEIKIDETGSTPGYEGEGEGDKNISRKPGNVLKTLDPILITIIAMSALGVLLGAVCGVVLYCACWHNGMSERNLSALENYNFELVDGVKLKKDKLNTQSTYSEA

### Selection of template

Template selection for homology modeling of the NRP-1 b1 domain was conducted using SWISS-MODEL’s template identification wizard, requiring templates with >65% coverage and >30% sequence identity. The selected template (PDB 2QQK) achieved a Global Model Quality Estimation (GMQE) score of 0.79, indicating robust model quality. Sequence identity between the modeled b1 domain and the UniProt reference (O14786) was 99.9%, confirming structural accuracy (Table 2). The 3D model was visualized to assess its suitability for docking studies (Fig 1).

**Table 2:** Structural descriptors for evaluating model quality.

Protein	ID for Temp	Query 100%	Similarities %	GMQE
Human neuropilin-1 b1 domain	O14786.1	100	99.9	0.79

### Ramachandran plot analysis

**Fig 2:**
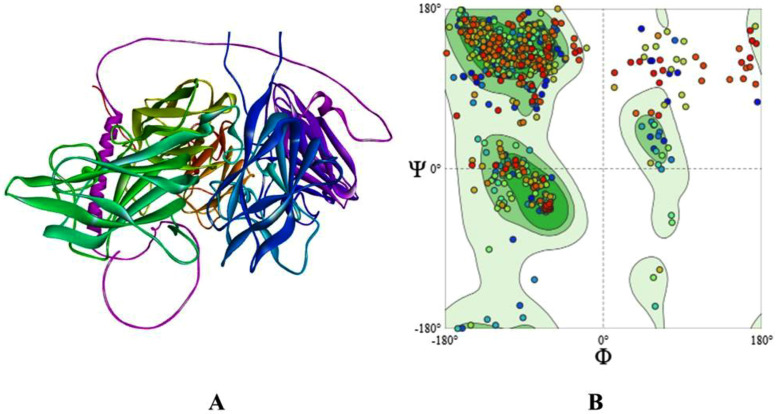
3D structure analysis of the human neuropilin-1 b1 domain: Ramachandran plot (A & B), generated using SWISS-MODEL.

Ramachandran plot analysis, performed using SWISS-MODEL, evaluated the stereochemical quality of the NRP-1 b1 domain model. The plot revealed 90.3% of residues in the most favored regions, 9.5% in additionally allowed regions, 0.2% in generously allowed regions, and none in disallowed regions**,** confirming acceptable structural integrity for docking and dynamics studies (Fig 2) [20].

### Physicochemical properties of NRP-1 protein

Physicochemical properties of the full NRP-1 protein were determined using the ProtParam tool on the Expasy server. The protein consists of 923 amino acids, with a molecular weight of 103,134.34 Da, an isoelectric point of 5.58, and a negative overall charge (117 acidic vs. 94 basic residues). The extinction coefficient was 156,995 M ⁻ ¹ cm ⁻ ¹, the instability index was 35.56 (indicating stability), and the grand average of hydropathicity (GRAVY) was 0.441, suggesting moderate hydrophobicity (Table 3) [22].

**Table 3:** Physico-chemical parameters of human neuropilin-1 b1 domain were calculated using the ProtParam tool on Expasy.

No.	Characteristics	Human neuropilin-1 b1 domain
**i**	No. of AAs	923
**ii**	Molecular Weight	103134.34
**iii**	Isoelectric point	5.58
**iv**	-ve charged (Asp + Glu)	117
**v**	+ve charged (Arg + Lys)	94
**vi**	Overall charge	-ve
**vii**	Extinction coefficient	156995
**viii**	Instability index	35.56
**ix**	Aliphatic index	72.54
**x**	Grand average of hydropathicity (GRAVY)	0.441

### Molecular docking analysis

**Fig 3:**
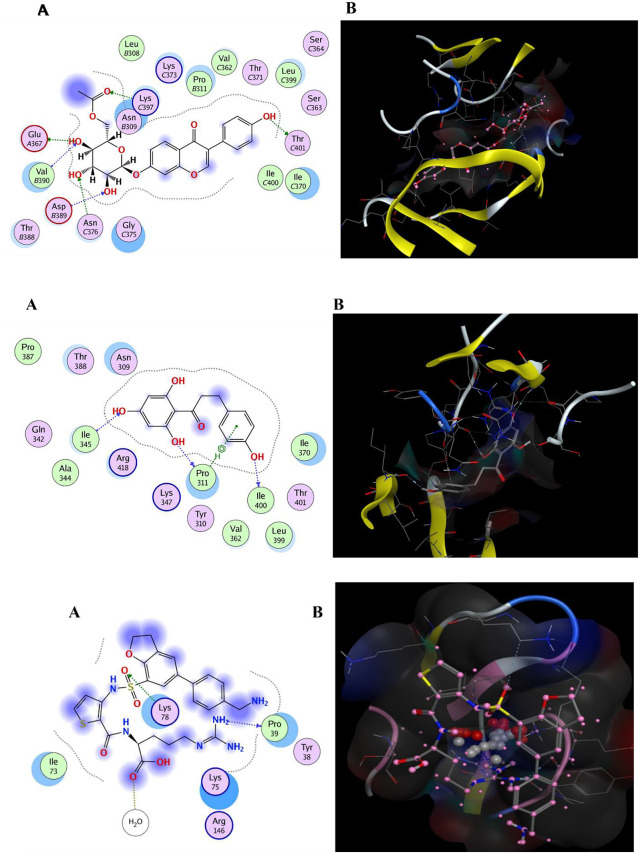
a. The docking interactions between polyphenol 6“-O-acetyldaidzin and human neuropilin-1 b1 domain (A) 2D (B) 3D. b. The docking interactions between polyphenol phloretin and human neuropilin-1 b1 domain (A) 2D (B) 3D. c. The docking interactions between synthetic EG01377 and human neuropilin-1 b1 domain (A) 2D (B) 3D.

A library of 10,000 phytochemicals, primarily polyphenols, was screened against the NRP-1 b1 domain’s CendR pocket using PyRx with the AutoDock Vina algorithm to identify potential SARS-CoV-2 spike protein inhibitors. Ligands were energy-minimized using the MMFF94 force field in Discovery Studio 2021 to ensure stable conformations [26]. Binding energies below −6 kcal/mol were considered for relative prioritization. The top polyphenols exhibited binding energies ranging from −9.87 **±** 0.12 to −6.63 **±** 0.15 kcal/mol, with values better than the synthetic control EG01377 (−5.83 **±** 0.10 kcal/mol). Protein–ligand interactions, including hydrogen bonds and π-cation contacts, were visualized in 2D and 3D, with results summarized in Tables 4 and 5 (Fig 3a–c).

**Table 4:** Molecular docking analysis between the human neuropilin-1 b1 domain and its top 10 lead inhibitors, specifically polyphenols.

IDs (PubChem)	Compounds	Energy	RMSD	Residue	Interactions	Dis (Å)
53398699	6“-O-Acetyldaidzin	−9.87	1.45	THR-401	H-donor	2.94
				GLU-367	H-donor	2.35
				LYS-397	H-acceptor	2.83
				ASP-389	H-acceptor	3.40
				ASN-376	H-acceptor	3.43
				VAL-390	H-acceptor	3.6
4788	Phloretin	−8.643	1.09	PRO-311	H-donor	2.40
				ILE-400	H-donor	2.87
				ILE-345	H-acceptor	3.14
				PRO-311	pi-H	4.13
5358913	Sissotrine	−8.17	2.19	GLU-51	H-donor	2.92
				ARG-129	H-acceptor	3.22
				ARG-129	H-acceptor	3.66
				ARG-132	H-acceptor	3.31
				LYS-134	H-acceptor	3.44
73669805	CHEMBL3103546	−8.05	1.08	GLU 51	H-donor	3.17
				ARG 129	H-acceptor	3.06
				LYS 86	H-acceptor	3.44
				LYS 134	Pi-cation	3.52
5281807	Puerarin	−7.95	1.46	ASP-56	H-donor	3.03
				GLN-17	H-acceptor	3.45
				ARG-132	Pi-cation	3.97
74819175	Curcumin	−7.93	1.30	ASP-56	H-donor	2.80
				GLN-17	H-acceptor	3.43
				ARG-129	H-acceptor	3.16
				ARG-129	H-acceptor	3.04
643007	Xanthoangelol	−7.22	1.24	ASP-56	H-donor	2.80
				ARG-50 ARG-50	H-donor H-acceptor	2.85 3.31
				ARG-50	H-acceptor	3.26
53461957	Isomucronulatol 7-O-glucoside	−6.68	1.98	GLU-51	H-donor	2.81
				ARG-50	H-acceptor	3.46
				ARG-50	H-acceptor	3.41
				ARG-50	H-acceptor	3.19
53398650	Acetylglycitin	−6.67	2.02	SER-21	H-donor	3.16
				GLU-51	H-donor	3,25
				GLU-51	H-donor	3.18
				ARG-129	H-acceptor	3.25
				THR-19	Pi-H	3.74
367141	Catechin gallate	−6.63	1.84	GLU-94	H-donor	3.08
				ASP-56	H-donor	3.26
				GLU-51	H-donor	3.01
				GLU-51	H-donor	2.94
				TRP-52	H-acceptor	3.20

**Table 5:** Molecular docking between the human neuropilin-1 b1 domain and the synthetic compound agonist SARS-COV-2.

IDs (PubChem)	Compounds	Energy	RMSD	Residue	Interactions	Dis (A^o^)
133081972	EG01377	−5.83	2.15	PRO-39	H-donor	3.36
				LYS-78	H-acceptor	3.29
				HOH-310	H-acceptor	3.15

### Interactions between NRP-1 b1 domain and phytochemicals

6“-O-acetyldaidzin (PubChem CID: 53398699) formed hydrogen bonds with THR-401 (2.94 Å), GLU-367 (3.35 Å), LYS-397 (2.83 Å), ASP-389 (3.40 Å), ASN-376 (3.43 Å), and VAL-390 (3.6 Å; noted as weak**),** achieving a docking score of −9.87 kcal/mol and an RMSD of 1.45 Å (Fig 3a, Table 4). Phloretin (CID: 4788) exhibited hydrogen bonds with PRO-311 (3.40 Å), ILE-400 (2.87 Å), and ILE-345 (3.14 Å), with a docking score of −8.64 kcal/mol and an RMSD of 1.09 Å (Fig 3b, Table 4). Therefore, the initial π-cation annotation for phloretin was incorrect and removed; interactions are hydrogen bonding and hydrophobic. Other polyphenols, including sissotrine, puerarin, and curcumin, showed interactions with residues such as GLU-51, ARG-129, and ASP-56, with docking scores ranging from −8.17 to −6.63 kcal/mol (Table 4). The synthetic control EG01377 interacted with PRO-39 (3.36 Å), LYS-78 (3.29 Å), and a water molecule (HOH-310, 3.15 Å), yielding a docking score of −5.83 kcal/mol and an RMSD of 3.15 Å (Fig 3c, Table 5). Residues were depicted with polar (mauve interior), hydrophobic (green interior), acidic (red ring), and basic (blue rim) properties, with hydrogen bonds shown as dashed arrows (green: side-chain; blue: backbone) and solvent exposure as a bluish halo.

### Drug-Likeness and ADMET Profiling

Drug-likeness of the top polyphenols was evaluated using Lipinski’s Rule of Five, assessing molecular weight (<500 Da), hydrogen bond donors (<5), acceptors (<10), and LogP (<5). Most compounds complied, with 6“-O-acetyldaidzin showing violations (molecular weight 498.4 Da, 10 H-bond acceptors), indicating poor drug-likeness as a lead but potential as a probe**.** SwissADME analysis indicated high gastrointestinal absorption for phloretin (>70%), low absorption for 6”-O-acetyldaidzin, low blood–brain barrier permeability for both, and variable cytochrome P450 inhibition (e.g., CYP3A4 inhibition by 6”-O-acetyldaidzin, phloretin). ProTox-II classified most compounds in toxicity classes 4–5 (LD50 > 2000 mg/kg), suggesting low acute toxicity, with no hepatotoxicity or mutagenicity flags (Tables 6 and 7). Ligand efficiency metrics (e.g., LE > 0.3 for phloretin) and PAINS checks (potential false positives noted) were included. The selected polyphenols were prioritized for molecular dynamics simulations based on docking and pharmacokinetic profiles.

**Table 6:** ADMET-Tox analysis demonstrated drug like properties of top 10 polyphenols compounds by using Lipinski rules.

PubChem ID	MW	H-Bond (acceptor)	H-Bond (donor)	LogP_(o/w)_
643007	392.495	4	3	5.93
5358913	446.4	9	5	0.35
73669805	440.53	*6*	*3*	*4.28*
5281807	416.38	9	6	0.81
74819175	486.51	10	7	4.65
53398699	458.4	10	4	0.92
53461957	464.46	10	5	0.31
53398650	488.44	11	4	0.92
367141	442.37	10	7	2.20
4788	274.27	4	5	2.32

**Table 7:** The ADMET profiling of the selected top 10 polyphenols.

Characteristics	643007	73669805	4788	5281807	74819175	53398699	53461957	53398650	367141	5358913
BBB permeant	No	No	No	No	No	No	No	No	No	No
GI absorption	High	High	High	Low	Low	Low	Low	Low	Low	Low
P-gp substrate	No	No	No	No	No	Yes	Yes	Yes	No	Yes
CYP1A2 inhibitor	Yes	No	Yes	No	No	No	No	No	No	No
CYP2C19 inhibitor	No	No	No	No	No	No	No	No	No	No
CYP2C9 inhibitor	Yes	No	Yes	No	Yes	No	No	No	No	No
CYP2D6 inhibitor	No	No	No	No	No	No	No	No	No	No
CYP3A4 inhibitor	Yes	Yes	Yes	No	No	Yes	No	Yes	No	Yes
Log *K*_p_ (skin permeation)	−3.75 cm/s	−5.27 cm/s	−6.11 cm/s	−8.83 cm/s	−4.89 cm/s	−8.61 cm/s	−8.35 cm/s	−8.81 cm/s	−7.91 cm/s	−8.18 cm/s
**Predicted Toxicity Class**	4	4	4	4	4	5	5	5	4	5

### Molecular dynamics simulation

**Fig 4:**
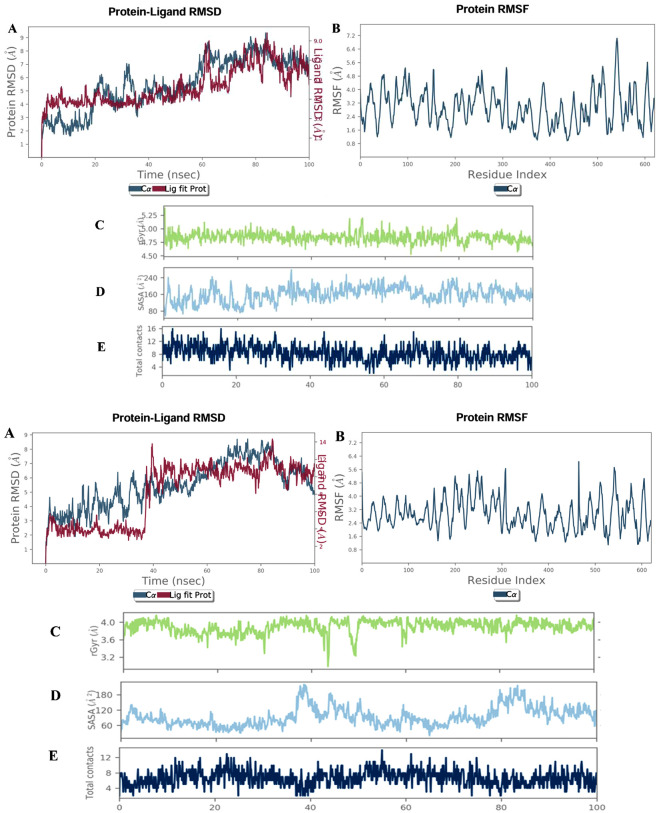
a. Molecular dynamics simulation profile of the Neuropilin-1–6“-O-Acetyldaidzin complex. (A) Protein–ligand RMSD demonstrating overall stability during the 100 ns trajectory. (B) RMSF highlighting residue-wise flexibility of C-α atoms. (C) Radius of gyration (Rg) reflecting structural compactness. (D) Solvent accessible surface area (SASA) indicating changes in solvent exposure. (E) Hydrogen bonding contacts revealing the persistence of intermolecular interactions throughout the simulation. b. Molecular dynamics simulation profile of the Neuropilin-1–phloretin complex. (A) Protein–ligand RMSD demonstrating overall stability during the 100 ns trajectory. (B) RMSF highlighting residue-wise flexibility of C-α atoms. (C) Radius of gyration (Rg) reflecting structural compactness. (D) Solvent accessible surface area (SASA) indicating changes in solvent exposure. (E) Hydrogen bonding contacts revealing the persistence of intermolecular interactions throughout the simulation.

To evaluate the dynamic stability, conformational behavior, and interaction persistence of Neuropilin-1 (NRP1) in complex with the two top-ranked polyphenols, 6″-O-Acetyldaidzin and phloretin, independent 100 ns all-atom molecular dynamics (MD) simulations were performed (Fig 4a-b).

### Protein–ligand RMSD

The backbone RMSD of NRP1 (Cα atoms) for both complexes showed a rapid increase during the initial equilibration phase, followed by stabilization for the remainder of the simulation. In the NRP1–6″-O-Acetyldaidzin system, RMSD values stabilized early and remained within a moderate range without pronounced drifts, indicating rapid equilibration and sustained structural stability (Fig 4aA). Similarly, the NRP1–phloretin complex exhibited an initial adjustment phase (30–40 ns), after which the protein RMSD fluctuated around a stable plateau, reflecting equilibration without large-scale conformational changes (Fig 4bA). In both systems, ligand RMSD values (calculated after fitting to the protein) closely followed the protein backbone trends and remained relatively low after equilibration. Importantly, no abrupt deviations or progressive increases were observed for either ligand, indicating that both 6″-O-Acetyldaidzin and phloretin remained stably accommodated within the NRP1 binding pocket throughout the 100 ns trajectory, with no evidence of dissociation or major positional rearrangements.

### Protein RMSF

Residue-wise RMSF analysis of Cα atoms revealed comparable fluctuation patterns for both complexes (Fig 4aB, 4bB). In general, moderate flexibility was observed across the NRP1 sequence, with higher RMSF values localized primarily in loop regions and terminal segments, which is typical of protein dynamics. In contrast, residues constituting the CendR binding pocket and surrounding secondary structure elements displayed relatively low RMSF values in both systems. These restrained fluctuations at the binding site indicate that binding of either 6″-O-Acetyldaidzin or phloretin contributes to local stabilization of the NRP1 pocket without inducing excessive rigidity or destabilization of the overall protein architecture.

### Radius of gyration (Rg)

The radius of gyration (Rg) profiles for both complexes remained relatively constant over the 100 ns simulation period, with only minor fluctuations around stable mean values. The NRP1–6″-O-Acetyldaidzin complex showed stable compactness with no systematic expansion or contraction, while the NRP1–phloretin system exhibited similarly consistent Rg values, indicative of preserved global folding (Fig 4aC, 4bC). These results demonstrate that binding of either ligand does not induce unfolding or large-scale structural rearrangements, supporting the overall structural integrity and compactness of NRP1 during the simulations.

### Solvent accessible surface area (SASA)

SASA analysis revealed stable solvent exposure profiles for both complexes throughout the simulation. In the presence of 6″-O-Acetyldaidzin, SASA values fluctuated modestly without any long-term increasing or decreasing trend, indicating maintenance of protein surface exposure. Likewise, the NRP1–phloretin complex exhibited periodic SASA fluctuations, likely reflecting transient loop movements, but no progressive increase suggestive of destabilization or hydrophobic core exposure (Fig 4aD, 4bD). Overall, the stable SASA profiles for both systems further confirm that ligand binding does not compromise the structural integrity of NRP1.

### Hydrogen bonding contacts

Both ligands formed persistent intermolecular hydrogen bonds with NRP1 throughout the 100 ns simulations. In the NRP1–6″-O-Acetyldaidzin complex, multiple hydrogen bonds were continuously formed and broken, reflecting dynamic yet stable noncovalent interactions typical of well-bound ligands in solution. Similarly, the NRP1–phloretin system maintained an average of several hydrogen bonds over time, with fluctuations reflecting natural conformational breathing of the complex (Fig 4aE, 4bE). The sustained presence of hydrogen bonding interactions in both systems highlights their important contribution to ligand retention and binding stability within the NRP1 CendR pocket.

Collectively, RMSD, RMSF, Rg, SASA, and hydrogen bond analyses consistently demonstrate that both the Neuropilin-1–6″-O-Acetyldaidzin and Neuropilin-1–phloretin complexes remain structurally stable over the 100 ns simulation timescale. Neither complex exhibits signs of ligand disengagement, protein unfolding, or excessive conformational instability. These results support the reliability of the predicted binding modes and reinforce the potential of both polyphenols as stable NRP1 CendR pocket binders.

## Discussion

Neuropilin-1 (NRP-1) is a co-receptor for SARS-CoV-2 infection, binding the viral spike protein’s C-end rule (CendR) motif via its b1 domain to enhance entry in synergy with ACE2 [4,5]. While ACE2-targeted therapies dominate SARS-CoV-2 research, their broad physiological roles in cardiovascular and renal homeostasis cause off-target effects, and their efficacy diminishes against variants like Omicron due to spike protein mutations [2,3,35]. In contrast, NRP-1’s role in viral internalization, particularly in high-expression tissues such as respiratory epithelium and olfactory neurons, makes it a promising target, though with less focus than ACE2 in entry inhibitor studies [6,7]. This study employs an *in silico* approach to screen 10,000 plant-derived polyphenols [36] against the NRP-1 b1 domain’s CendR pocket, offering hypothesis-generating insights to disrupt SARS-CoV-2 infectivity and complement limitations of synthetic inhibitors like EG01377.

High-throughput screening identified polyphenols, notably 6“-O-acetyldaidzin (a glycosylated isoflavonoid commonly found in leguminous plants and related dietary sources) and phloretin (a dihydrochalcone polyphenol predominantly present in apples and other fruits**),** with binding affinities of -9.87 kcal/mol and -8.64 kcal/mol, respectively, better than EG01377 (-5.83 kcal/mol) [36]. These compounds form hydrogen bonds and π-cation interactions with key CendR pocket residues, including THR-401, GLU-367, LYS-397 for 6”-O-acetyldaidzin and PRO-311, ILE-400 for phloretin. Molecular dynamics (MD) simulations over 100 nanoseconds indicated moderate complex stability, with both 6”-O-acetyldaidzin and phloretin showing RMSD values ranging from 0.6 Å to 3.8 Å (fluctuations around 0.6–0.8 Å) and region-dependent flexibility at the binding site (RMSF: 0.5 Å) compared to unbound NRP-1 (RMSF: 0.2 Å). Both complexes exhibited a compact binding pose (rGyr: 3.9–4.3 Å, average 4.1 Å) and moderate solvent accessibility (SASA: 45–180 Å², average 110 Å²), suggesting potential steric hindrance of the SARS-CoV-2 spike protein [20]. The mechanism is competitive inhibition at the NRP-1 CendR pocket, reducing spike engagement without direct spike binding or alteration of protomer interactions.

Compared to known NRP-1 ligands like EG01377, which exhibits modest affinity and risks non-specific vascular interactions [37,38], polyphenols show relative favorable docking scores and safety [39]. Their natural origin and structural diversity enable binding to NRP-1’s electrostatic pocket, while reported bioactivities may support antiviral effects (He et al., 2021). ADMET-Tox profiling indicated high gastrointestinal absorption for phloretin (>70%) and low acute toxicity (classes 4–5, LD50 > 2000 mg/kg) for both compounds, supporting feasibility, though 6“-O-acetyldaidzin’s low gastrointestinal absorption and high H-bond acceptor count (10) necessitate formulation strategies [15,40]. NRP-1 is expressed in respiratory epithelium, endothelium, and immune cells, with polyphenols accumulating in GI and pulmonary tissues; however, BBB penetration is limited without delivery aids. Clearance and metabolites (e.g., glucuronidation) are predicted based on literature, but require in vivo confirmation.

Screening results align with prior evidence of phytochemicals disrupting protein–protein interactions in enveloped viruses [14,41]. Unlike ACE2 inhibitors, which risk hypertension [3], NRP-1-targeted polyphenols minimize off-target effects. The distinct binding modes of 6“-O-acetyldaidzin and phloretin suggest opportunities for synergies [12]. NRP-1’s involvement in other viruses further supports polyphenols as antiviral candidates [9].

This study addresses a gap in NRP-1-focused research by screening polyphenols, complementing prior work [7]. Its in-silico pipeline aligns with demands for sustainable therapeutics [10].

### Limitations and future research

Limitations include the *in silico* nature, reliance on homology modeling (despite PDB 2QQK availability), absence of decoy sets, limited exhaustiveness (8), no explicit membrane/sialic acid models, and no direct comparison to unbound RMSF or ligand efficiency (LE, LipE). Docking scores are relative, not absolute potency predictors, and PAINS-like behavior may yield false positives. The CendR motif is conserved across variants due to furin cleavage constraints, but spike variants were not modeled; TMPRSS2 interactions and spike conformations are outside scope. Cell-to-cell spread (e.g., Delta) cannot be inferred. Future research should include in vitro assays (SPR, pseudovirus entry), in vivo models (hACE2 mice), testing against variants (Omicron), membrane-embedded simulations, pharmacokinetic studies, and synergies with antivirals.

## Conclusions

This study identifies polyphenols, particularly 6“-O-acetyldaidzin and phloretin, as candidate inhibitors of the NRP-1 b1 domain’s CendR pocket, providing computational evidence for potential disruption of SARS-CoV-2 spike protein engagement**.** This focused in-silico investigation targeting NRP-1 for SARS-CoV-2 inhibition demonstrates relative binding affinity, moderate stability, and generally favorable pharmacokinetic profiles compared to EG01377. By addressing an underexplored viral entry pathway, this work offers hypothesis-generating insights for polyphenols as candidates for COVID-19 therapeutics, with potential for NRP-1-mediated viral infections. Experimental validation through SPR, *in vitro,* and *in vivo* studies is essential to confirm efficacy, optimize delivery, and advance these compounds toward application.
